# The cerebellum in dual-task performance in Parkinson’s disease

**DOI:** 10.1038/srep45662

**Published:** 2017-03-30

**Authors:** Linlin Gao, Jiarong Zhang, Yanan Hou, Mark Hallett, Piu Chan, Tao Wu

**Affiliations:** 1 Department of Neurobiology, Key Laboratory on Neurodegenerative Disorders of Ministry of Education, Beijing Institute of Geriatrics, Xuanwu Hospital, Capital Medical University, Beijing, China; 2 Beijing Key Laboratory on Parkinson’s Disease, Parkinson Disease Center of Beijing Institute for Brain Disorders, Beijing, China; 3 Human Motor Control Section, Medical Neurology Branch, National Institute of Neurological Disorders and Stroke, National Institutes of Health, Bethesda, MD, USA

## Abstract

Parkinson’s disease (PD) patients have difficulty in performing a dual-task. It has been suggested that the cerebellum is important in dual-tasking. We used functional MRI to investigate the role of the cerebellum in performing a dual motor and cognitive task in PD patients. We have examined whether there are any areas additionally activated for dual-task performance, and compared the neural activity and functional connectivity pattern in the cerebellum between PD patients and healthy controls. We found that the right cerebellar vermis and left lobule V of cerebellar anterior lobe were additionally activated for dual-task performance in healthy controls and for motor task in PD patients. We didn’t find any cerebellar regions additionally activated while performing dual-task in PD patients. In addition, the right cerebellar vermis had enhanced connectivity with motor and cognitive associated networks in PD patients. PD patients have limited cerebellar resources that are already utilized for single tasks and, for dual tasks, cannot augment as necessary in order to integrate motor and cognitive networks.

Parkinson’s disease (PD) patients commonly have difficulties in performing two tasks simultaneously^1^,^2^. This problem can be observed in performing two motor tasks, two cognitive tasks or combined cognitive and motor tasks at the same time^3^,^4^. It has been shown that dual-task performance is significantly associated to motor problems in PD, like bradykinesia^5^, falling^6^, and freezing of gait^7^. However, the neural reasons contributing to this problem remain unclear, and have been suggested relating to limited attentional resources^3^, defective central executive function and less automaticity^8^,^9^.

By now, only a small number of neuroimaging studies have investigated the neural correlates underlying dual-tasking in PD^8^,^10^,^11^. When performing dual-tasks combining a sequential finger movement and a counting task, the precuneus was additionally activated compared with the single component tasks in PD and healthy controls. PD patients had enhanced activity in the cerebellum, premotor cortex (PMC), parietal cortex, precuneus and prefrontal cortex compared to the controls^8^. A few recent studies have investigated dual-tasking related functional or structural connectivity in PD patients with freezing of gait. One showed that worse dual-task performance was associated with decreased functional connectivity within the striatum and between the caudate and superior temporal lobe, and with increased connectivity between the dorsal putamen and precuneus^11^. In another study, dual-task interference was correlated with asymmetry of pedunculopontine nucleus structural connectivity in PD patients^10^.

In a recent study, using a dual-task paradigm with a simple motor task and a cognitive (counting) task, we found that the cerebellar vermis and lobule V of the cerebellar anterior lobe are additionally activated for dual-task in healthy subjects, and these cerebellar regions had functional connectivity with extensive motor- and cognitive-related regions^12^. It is likely that the cerebellum is involved in integrating motor and cognitive networks in order to perform dual motor- cognitive tasks properly. The role of the cerebellum in PD has been long overlooked. However, in recent years, it has been recognized that there are reciprocal anatomical connections between the cerebellum and basal ganglia^13^. Moreover, increasing evidence has suggested that the cerebellum has PD-related structural and functional modulations and may contribute substantially to the clinical symptoms of PD^14^. Therefore, in the current study, we used functional MRI (fMRI) to investigate the neural activity and network connectivity in the cerebellum during execution of dual-task in PD patients. We hypothesized that there is no cerebellar region additionally activated, and the connectivity between the cerebellum and motor and cognitive associated networks is changed while performing dual-task in PD patients. The dysfunction of the cerebellum might be a reason contributing to the difficulty in dual-tasking in PD.

## Results

### Task performance

During fMRI scanning, both groups had no errors in performing the single and dual-tasks. The data were normally distributed. As the accuracy was 100% in performing all single and dual-tasks, there was no difference of performance between the dual-task and the single tasks within each group, and no difference of performance of the dual-task between the two groups (repeated-measures ANOVA, p = 1). Interference scores were calculated by taking the difference between the accuracy for the single task and dual-task, and then dividing this difference by the accuracy in the single task for each subjects. As there was no difference of accuracy between the single and dual-tasks, the interference scores were 0.

### FMRI results

#### Brain activity

**Figure 1:**
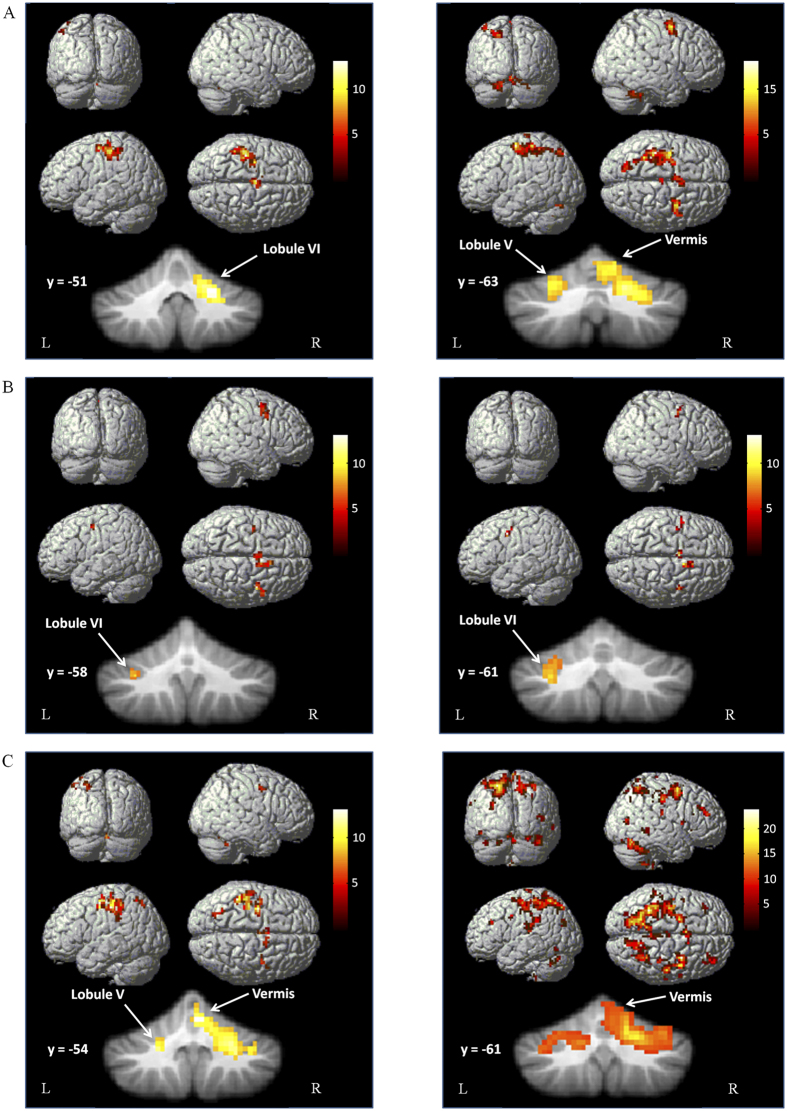
Brain activity in PD patients and controls. Brain regions activated during performing the right hand finger tapping task (**A**), counting task (**B**) and dual-task (**C**) in the healthy control group (left column), and in PD patients (right column). One sample t-test, P < 0.05, FWE corrected. T-value bars are shown on the right.

Performance of the single tapping task was associated with activation in the left primary sensorimotor cortex (M1), caudal supplementary motor area (SMA-proper), and left premotor cortex (PMC) in both groups (one sample t-test, P < 0.05, FWE corrected). Bilateral cerebellum was activated in PD patients, while only the right lobule VI of cerebellum was activated in controls (Fig. 1A). The counting task activated the rostral SMA (pre-SMA), bilateral PMC, and left lobule VI of cerebellum in both PD and control subjects (Fig. 1B). During performance of the dual-task, both groups had activations in the left M1, pre-SMA, SMA-proper, bilateral PMC, bilateral parietal cortex, precuneus, and bilateral cerebellum, while PD patients additionally activated bilateral prefrontal cortex (Fig. 1C).

**Figure 2:**
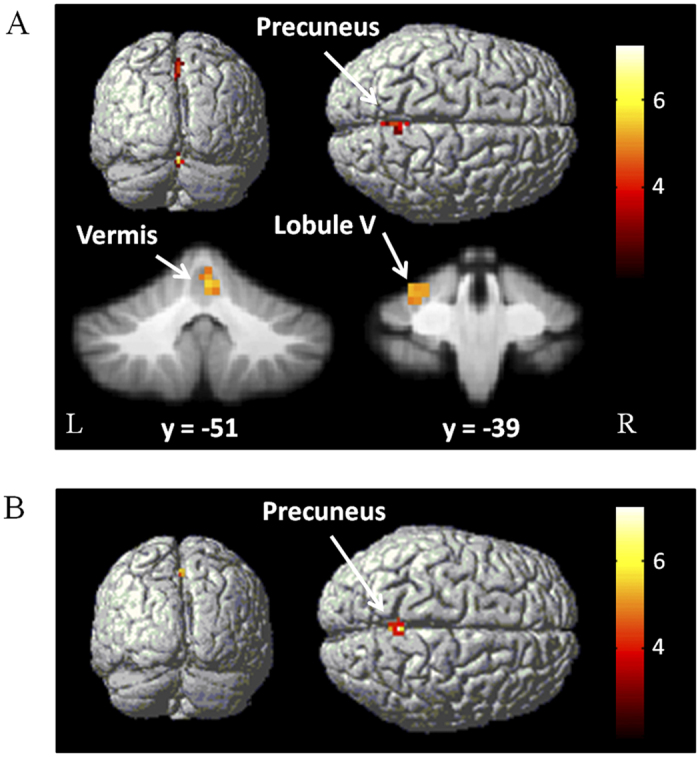
Brain regions additionally activated in the dual-task. Brain areas more activated in the dual-task compared with single component tasks in controls (**A**), and in PD patients (**B**). One-way ANOVA, P < 0.05, FWE corrected. T-value bars are shown on the right.

Compared with the single component tasks, dual-task performance additionally activated the RVM, LCV, and precuneus in controls; in contrast, the precuneus was the only additionally activated area in PD patients (one-way ANOVA, P < 0.05, FWE corrected; Fig. 2, Table 1).

**Figure 3:**
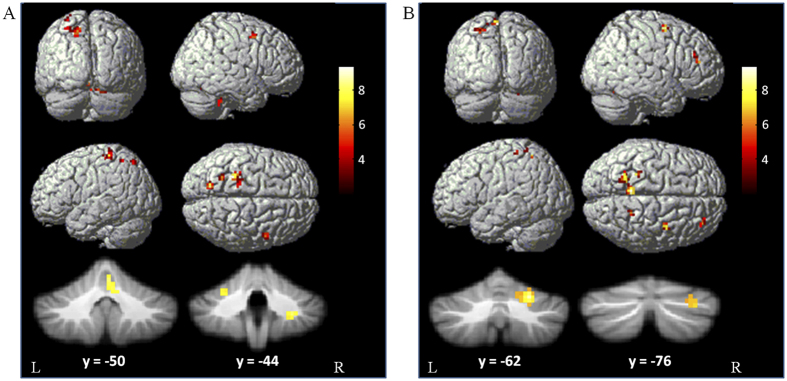
Brain areas more activated in PD patients. Brain areas more activated in PD patients than in controls during performing tapping task (**A**), and dual-task (**B**). Two-sample t-test, P < 0.05, FWE corrected. T-value bars are shown on the right.

Compared with the controls, PD patients had more activation in the left M1, right PMC, left parietal cortex, and bilateral cerebellum during performance of the tapping task, and had more activation in the right prefrontal cortex, right PMC, bilateral parietal cortex, bilateral precuneus, and right cerebellum while performing the dual-task (two sample t-test, p < 0.05, FWE corrected; Fig. 3, Table 2). There was no difference between the groups in performing the counting task. We did not detect more activity in controls than in patients in any region while performing the tapping, counting, or dual-task (two sample t-test, P < 0.05, FWE corrected).

#### Brain deactivation

During performance of dual-task, there was deactivation in the precuneus, left inferior parietal lobule and left middle temporal gyrus in healthy controls. In PD patients, there was deactivation in the precuneus, left inferior parietal lobule and left middle frontal gyrus (Supplementary Fig. 1; one sample t-test, P < 0.05, FWE corrected). There was no significant between-group difference of deactivation while performing these tasks.

#### Network connectivity

##### Connectivity in the RVM

**Figure 4:**
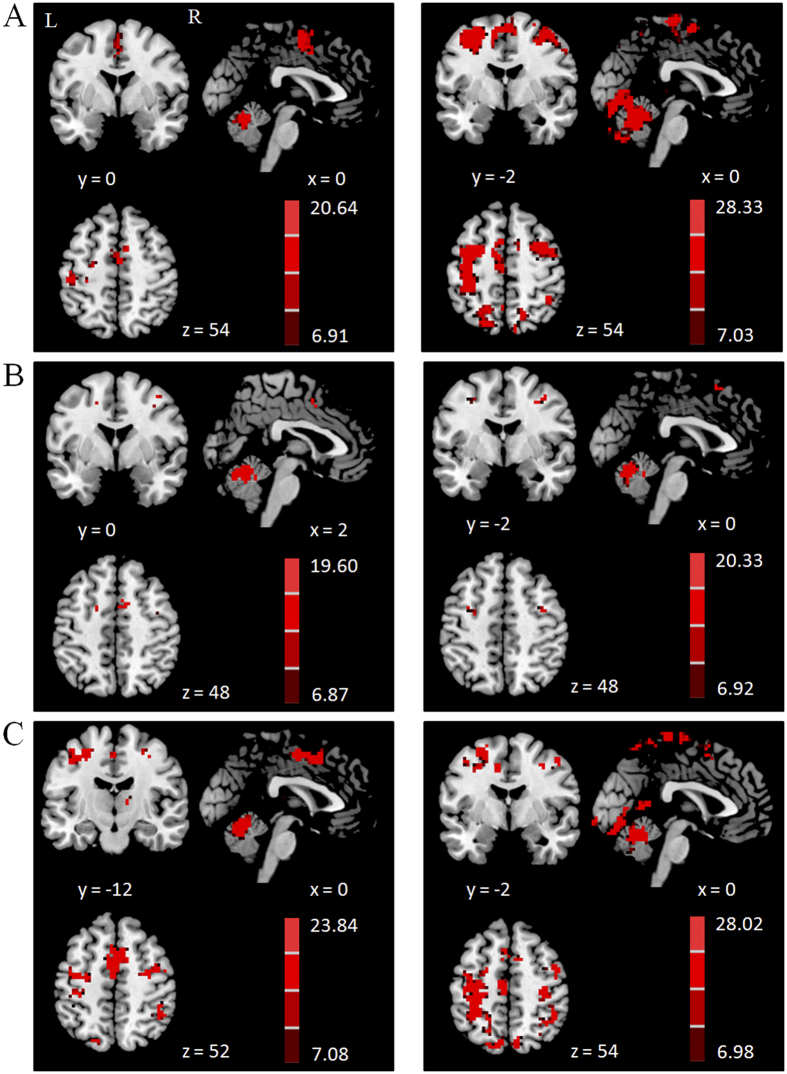
Brain regions connected with the right vermis of the cerebellum (RVM). Brain regions functionally connected with the RVM during performing the right hand finger tapping task (**A**), counting task (**B**) and dual-task (**C**) in the healthy control group (left column), and in PD patients (right column). One sample t-test, P < 0.05, FWE corrected. T-value bars are shown on the right.

In healthy controls, the RVM functionally connected with the bilateral cerebellum, left M1, left PMC, and SMA-proper while performing the finger tapping task, and had connectivity with the bilateral cerebellum, pre-SMA, and bilateral PMC when performing the counting task. During performance of the dual-task, the RVM connected with the bilateral cerebellum, left M1, bilateral PMC, pre-SMA, SMA-proper, bilateral parietal cortex, and right thalamus (Fig. 4, Supplementary Table 3).

In PD patients, the RVM functionally connected with the bilateral cerebellum, left M1, bilateral PMC, pre-SMA, SMA-proper, right prefrontal cortex and bilateral parietal cortex while performing the finger tapping task, and had connectivity with the bilateral cerebellum, pre-SMA, and bilateral PMC when performing the counting task. During performance of the dual-task, the RVM connected with bilateral cerebellum, bilateral M1, bilateral PMC, pre-SMA, SMA-proper, bilateral temporal lobe, bilateral parietal cortex, and left thalamus (Fig. 4, Supplementary Table 2).

**Figure 5:**
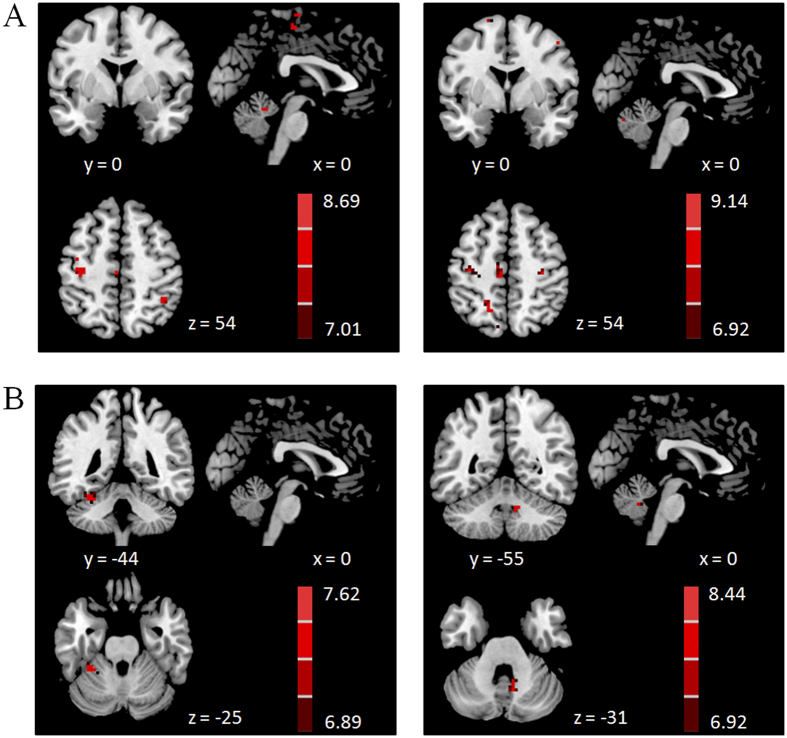
Brain regions more connected with the ROIs in PD patients. Brain areas more connected with the RVM (**A**) or LCV (**B**) in PD patients than in controls during performing tapping task (left column), and dual-task (right column). Two-sample t-test, P < 0.05, FWE corrected. T-value bars are shown on the right.

Between-group comparisons showed that in performance of the tapping task, the RVM had enhanced connectivity with the left M1, bilateral cerebellum, right inferior parietal lobule and left paracentral lobule in PD patients. During performing the dual-task, the RVM had increased connectivity with the bilateral M1, right cerebellum, left superior parietal lobule, and left paracentral lobule in PD patients (Fig. 5A). There was no between-group difference while performing the counting task.

##### Connectivity in the LCV

In both groups, the LCV had functional connectivity with the posterior lobe of the bilateral cerebellum, SMA-proper, and PMC during execution of the single tapping task. In performing the single counting task, the LCV had connectivity with the bilateral cerebellum, pre-SMA, and PMC. During performance of the dual-task, the LCV showed connectivity with the bilateral cerebellum, pre-SMA, SMA-proper, PMC, and parietal cortex (Supplementary Fig. 2, Supplementary Tables 3 and 4).

The between-group comparison showed that the LCV had enhanced connectivity with the left anterior lobe of cerebellum during performing the tapping task, and had increased connectivity with the right anterior lobe of cerebellum in performance of the dual-task in PD patients compared to controls (Fig. 5B). There was no between-group difference while performing the counting task.

We did not find any brain regions additionally connected with the RVM or LCV in dual-task compared with the single component tasks in both groups.

##### Connectivity in the precuneus

The results of connectivity in the precuneus during performance of dual-task were showed in Supplementary Fig. 3. The between-group comparison showed that the precuneus had enhanced connectivity with the right anterior lobe of cerebellum, right anterior cingulate gyrus, and left superior parietal lobule in PD patients compared to controls (Supplementary Fig. 3C; two sample t-test, P < 0.05, FWE corrected). We did not find increased connectivity in controls than in patients.

## Discussion

In the current study, we investigated the role of the cerebellum in dual-tasking in PD patients. We found that the right cerebellar vermis and left lobule V of cerebellar anterior lobe were additionally activated for execution of a dual motor and cognitive task in healthy controls. In contrast, there were not any cerebellar regions additionally activated while performing this dual-task in PD patients. In addition, the dual-tasking related neural connectivity pattern was different between PD patients and controls. Our findings suggest that the cerebellum might play a role in dual-tasking deficiency in PD.

When people attempt to perform two tasks at the same time, impaired performance tends to occur, which is defined as dual-task interference. In the current study, most subjects did not show significant dual-task interference after practice, which supports previous findings that practice can diminish dual-task interference^8^,^12^,^15^,^16^,^17^,^18^,^19^,^20^. While five patients could not perform the dual-task properly even after training, most patients performed the dual-task at the same level as that in controls. This finding is consistent with previous observations that PD patients have more difficulty in performing dual-task; however, they can perform some relatively simple dual-tasks correctly after training^8^,^20^.

In agreement with our previous study^12^, we found that in healthy subjects, the RVM and LCV were additionally activated in performing a dual-task (combining a motor task and a cognitive counting task) compared with single tasks (Fig. 2). Whether the cerebellum is also involved in other types of dual-tasks would need further investigation. In contrast, no regions in the cerebellum were additionally activated in the dual-task in PD patients. We found that during performance of the single tapping task, the RVM and LCV were already activated in PD patients (Fig. 1A), and were more activated in PD patients than in controls (Fig. 3A). Hyperactivation in the cerebellum in PD patients has been extensively reported^20^,^21^,^22^,^23^,^24^. However, the nature of this phenomenon remains unclear. A common explanation is that it is a reflection of the compensatory effect^14^,^20^,^24^. Performance of a dual-task with high accuracy indicates that the component tasks are performed automatically^25^,^26^. It is known that PD patients have great difficulty in performing movements automatically, and require more brain activity, which might be to compensate for basal ganglia dysfunction in order to perform automatic movements^20^. However, more activation in the cerebellum might also represent a primary pathophysiological change of PD^14^, as a consequence of the inability to inhibit contextually inappropriate circuits secondary to abnormal basal ganglia outflow^27^. Our findings indicate that due to either compensatory or pathological effects, the RVM and LCV were already activated in the single tapping task. Presumably, the limitation of neural resources in these cerebellar regions has been reached even with single tasks in PD patients, and these regions could not be further recruited in dual tasks.

Functional connectivity reflects the integration within functionally specialized networks for a given task^28^. In healthy controls, the RVM and LCV mainly connected with motor-related regions, e.g. the M1 and SMA-proper, during performance of the tapping task. In contrast, these regions had more connections with cognitive-related areas, e.g. the pre-SMA, while performing the counting-task. The SMA-proper is primarily involved in movement behaviors, while the pre-SMA is associated with cognitive behaviors^29^,^30^,^31^,^32^,^33^. The pre-SMA is certainly also involved in motor behavior. However, anatomical and physiological evidence suggests that the pre-SMA is more like a prefrontal area than a motor area, and provides cognitive, sensory or motivational inputs for motor behavior^34^. In performing the dual-task, the RVM and LCV had connectivity with both motor and cognitive-related areas (e.g. SMA-proper and pre-SMA), but did not connect to any regions that were not connected in the single component tasks. Our previous study found that the RVM and LCV had strengthened connectivity with motor and cognitive networks during the practice of dual-task^12^. These findings suggest that the role of the RVM and LCV is likely to integrate the motor and cognitive networks, and may also adjust these networks to be more efficient in order to perform the dual motor and cognitive task properly^12^.

In PD patients, the pattern of network connectivity in the RVM and LCV was similar with that in controls while performing the counting task. In contrast, the pattern of connectivity while performance of the single tapping task was different between the two groups, especially in the RVM. The RVM had connections not only with the motor-related regions, but also with the pre-SMA and prefrontal cortex. The prefrontal cortex is known to be involved in generating new movements^35^,^36^, task rehearsal^37^, attention to action^9^,^38^, and in monitoring of motor performance^39^,^40^. In addition, the connectivity between the RVM and motor-related areas, e.g. the M1, was increased in PD patients. As a consequence, in PD patients, it might be difficult for the RVM to recruit more motor, cognitive and attentional associated networks for the dual-task.

These cerebellar regions also showed enhanced connectivity during performance of the dual-task, which is possibly a compensatory effort^41^,^42^,^43^. However, the modulation of network connectivity might be a reflection of pathological effects. In healthy subjects, these cerebellar regions have distinct connectivity profiles while performing different tasks. The re-organization of networks may reduce the segregation of neural circuits, and may disrupt the balance between the networks. A recent study found that in PD patients with freezing of gait, the worse dual-task performance was correlated with the altered balance between cognitive and motor networks^11^. Presumably, the modulated connectivity profile and disrupted balance between the motor and cognitive networks might result in the difficulty of these cerebellar regions in integrating these networks while performing dual-tasks.

The enhanced activity and connectivity also indicate that the efficiency of neural systems is reduced in PD patients. The recruitment of more brain resources can help maintain the performance of some simple dual-tasks. However, when performing more complex dual-task, the limitation of brain capacity might be exceeded. This might be a reason contributing to the difficulty in performing the more complex dual-tasks in PD patients.

Besides the cerebellar regions, the precuneus was additionally activated in the dual-task compared to the single component tasks in both groups. This finding is consistent with previous reports on healthy controls^12^,^44^,^45^ and PD patients^8^. The precuneus has been associated with attention^46^, orientation, monitoring^47^, working memory^48^ and preparation of movements^49^. It has been suggested that the additionally activated precuneus in performing dual-tasks is likely due to increased demands of attention than in performing single tasks^44^. A recent study showed that in PD patients with freezing of gait, worse dual-task performance was correlated with increased connectivity between the precuneus and dorsal putamen^11^. We found that the precuneus had enhanced connectivity with some areas in PD patients compared to controls. Whether the precuneus plays a substantial role in dual-task deficiency in PD needs to be clarified in future.

There were some limitations in the current study. First, we used a relatively low-resolution fMRI scanning protocol. In recent years, some high-resolution fMRI (e.g. 1.5 mm^3^ in plane resolution) scanning protocols have been developed. Future studies with high-resolution fMRI scanning will be helpful to investigation on cerebellum. Second, we applied a relatively easy paradigm to investigate dual-task related neural correlates. This paradigm is working well in distinguishing dual-task related activity from single task activations^12^. However, as PD patients also can perform this dual-task correctly after training, this paradigm may not sensitive to detect dual-task deficiency in PD. An improved paradigm might be developed in future. Third, as head motion has significant impact on connectivity results^50^, we include head motion parameters as regressors to minimize the influence of head motion on our results. However, as head motion may be task-correlated, whether head motion correlated with the task behaviors should be examined. As we only measured the correction of finger tapping and counting, this correlation analysis cannot be performed in the current study, which needs to be examined in future studies. Forth, we chose patients at a relatively mild stage to ensure that most patients could perform the dual-task properly after training. It is possible that PD patients at more advanced stage have more difficulty in performing dual-task, and neural changes in more advanced patients may be different from that in patients at mild stage. Moreover, we only focused on the cerebellum in the current study. It has been shown that some cortical regions (e.g. the SMA and PMC) are also involved in dual-task deficiency in PD^8^,^10^,^11^,^51^. In addition, several studies have shown that the dorsolateral prefrontal cortex (DLPFC) is involved in dual-tasking^52^,^53^. Therefore, the cerebellum is likely one of the reasons contributing to dual-task deficiency in PD. More studies are warranted to fully understand this problem.

In conclusion, the present study found that the right cerebellar vermis and left lobule V of cerebellar anterior lobe were additionally activated for dual-task performance in healthy controls. In contrast, there was no cerebellar region additionally activated while performing dual-task in PD patients. In addition, these cerebellar regions had enhanced connectivity with motor and cognitive associated networks in PD patients. Our findings suggest that the limited cerebellar resources and the difficulty of these cerebellar regions in integrating motor and cognitive networks might contribute to dual-tasking deficiency in PD patients.

## Methods

### Subjects

Twenty-five PD patients were involved in this study. The diagnosis of PD was based on the UK Parkinson’s Disease Society Brain Bank Clinical Diagnostic Criteria^54^. Patients were assessed with the Unified Parkinson’s Disease Rating Scale (UPDRS)^55^, the Hoehn and Yahr disability scale^56^, and Mini-Mental State Examination while off their medications. Bradykinesia/rigidity was the predominant symptom and was more severe on the right side in every patient. To avoid disturbance of the fMRI signal, all patients were chosen to have at most a mild tremor (no more than mild degree in the items 3.15 to 3.18 of the UPDRS examination). Twenty age and gender matched healthy subjects were recruited as the control group. All patients and control subjects were right-handed according to the Edinburgh Inventory^57^. Five patients were excluded because they could not perform the dual-task properly. In addition, two patients and two controls had excessive head motion during the fMRI acquisition, and their datasets were discarded. The demographics and clinical details from the remaining controls and patients are shown in Table 3. The experiments were performed according to the Declaration of Helsinki and were approved by the Institutional Review Board of Xuanwu Hospital. All subjects gave their written informed consent for the study.

### Task

All subjects were asked to perform two single tasks and one dual-task. The paradigm was similar to that in a recent study^12^. Single tasks contained a motor task and a number counting (cognitive) task. The motor task was a self-paced tapping task in which subjects briskly tapped their right index and middle fingers alternatively at 1 Hz frequency, with amplitude of about 2.5 cm. For the counting task, a random series of the numbers 2, 3, 5, and 8 were presented on a screen and subjects were asked to identify the number of times they saw a specified target number. The numbers were presented at an irregular interval (average interval 1.5 s). In the dual-task, subjects performed the tapping and counting tasks simultaneously. As a first priority, we asked the subjects to perform the motor task correctly, and then a second priority, to count the target numbers correctly.

Before the fMRI scanning, subjects were given one hour practice in order to perform the single and dual-tasks correctly. The successful performance of the dual-task was determined by the execution of three dual-task trials in a row without errors (each trial lasted 3 min), and without behavioral difference from the single tasks. As mentioned above, five patients were excluded because they could not perform the dual-task properly after one hour’s practice.

### fMRI Procedure

fMRIs were performed on a 3 T MR scanner (Trio system; Siemens Magnetom scanner, Erlangen, Germany). A standard head coil was used with foam padding to restrict head motion. All subjects underwent high- resolution MRI scanning, which included a 3D T1-weighted magnetization prepared rapid acquisition gradient echo (MPRAGE) scan (TR = 1600 ms, TE = 2.13 ms, 176 sagittal slices, slice thickness = 1.0 mm, field of view (FOV) = 224 mm × 256 mm, voxel size of 1 mm^3^ isotropic), and a T2-weighted MRI scan (TR = 5000 ms, TE = 87 ms, 35 axial slices, slice thickness  = 4 mm, FOV = 256 mm × 256 mm, voxel size = 1 × 1 × 4 mm^3^). Blood-oxygen-level dependent data were acquired with gradient-echo echo-planar sequences (TR = 2000 ms, TE = 40 ms, 33 axial slices, slice thickness = 3.5 mm, no gap, Flip angle = 90°, FOV = 256 mm × 256 mm, matrix size = 64 × 64). As it has been shown that heart rate has influence on fMRI signal^58^, we monitored heart rate in each subject during fMRI scanning.

An electrical response device that could work inside the MRI scanner was fixed to each subject’s right hand. The response device had 2 buttons, corresponding to the index and middle fingers of the right hand and was used to record finger movements during fMRI scanning. For each subject, there were three runs in fMRI scanning session. Each run was 8 min. All runs were block-designed and contained two conditions, which were defined as the “rest” and “task” condition, respectively. Each condition lasted 30 s and was repeated eight times. In the rest condition, subjects were asked to relax and focus on the screen in front of them without moving or thinking while inside the scanner. The task condition in each run contained one of the right hand tapping, number counting, or dual-tasks. Visual signals were presented on the screen to inform the subjects to switch from rest to task condition and vice versa. The subjects viewed the visual signals through a mirror built into the head coil. Task order of these three runs was randomized across subjects in each session. After scanning each run, subjects were asked to report the whole number of the target number in that run.

### Behavioral data analysis

The behavioral measurements included the accuracy and frequency for finger-tapping, and number of target numbers in each run. Wrong button presses for motor task or incorrect numbers being reported for the counting task were considered errors. The accuracy in each single and dual-task were entered into a repeated-measures ANOVA to calculate the within- and between-group differences in performance between single and dual-tasks.

### Imaging data analysis

#### Data Preprocessing

Image analysis was performed with SPM8 software (Wellcome Institute of Cognitive Neurology, London, UK). fMRI data were slice-time corrected and aligned to the first image of each run for motion correction. After spatial normalization, images were resampled into voxels that were 3 × 3 × 3 mm in size, and smoothed with a 6 mm Gaussian smoothing kernel. Each participant’s movement parameters were examined. As described previously, two patients and two controls had excessive head motion (more than 1 mm maximum translation in x, y or z, or 1° of maximum angular rotation about each axis), and their datasets were discarded.

#### Brain Activity Analysis

Data were first analyzed for each single participant separately on a voxel-by-voxel basis using the general linear model approach for the time series. We defined a model using a fixed effect boxcar design convolved with a hemodynamic response function for analysis of task-dependent activation. We added the head motion parameters as regressors to optimally control for the motion effects. In addition, heart rate was used as a regressor to reduce its influence on activation in the cerebellum^58^. At the first-level, a contrast representing the effect of the “task” condition compared with the “rest” condition was calculated in each participant. These contrast images were used in the second level for random-effects analyses.

At the second level, first, a one-sample t-test model was used to identify the brain activity in each condition in each group. Then, we used one-way ANOVA to explore the brain regions that were additionally activated in the dual-task compared to single tasks in each group (dual-task–tapping–counting)^8^,^12^,^59^. Additionally, a two-sample t-test model was used to explore the difference between patients and normal subjects in each condition.

#### Brain Activity in the Cerebellum

The activations specific to the cerebellum were further analyzed with the Spatially Unbiased Infra-tentorial Template toolbox (SUIT Version 3.2)^60^,^61^. We used the high resolution T1- images to isolate the cerebellum from the rest of the brain, and normalized to the SUIT template using a nonlinear deformation. Then, we used unsmoothed functional data to calculate the contrasts in each subject and in each condition with the same procedures for the whole brain activity analysis. These contrast images were resliced using the deformation found in the last step and smoothed. At the second level, the activations in the cerebellum in each condition were analyzed. The results were overlaid on the high-resolution SUIT atlas of the human cerebellum^60^,^61^.

#### Brain Deactivation Analysis

In addition, we analyzed deactivation during performance of the dual-task. The deactivation means more voxel intensity in the “rest” condition than that in the “task” condition. The deactivation in each group was measured in each group by a one-sample t-test. Then, a two-sample t-test model was used to explore the difference of deactivation between the groups.

#### Network Connectivity Analysis

According to the results from brain activity analysis in this study, the right cerebellar vermis (RVM), left lobule V of the cerebellar anterior lobe (LCV) and precuneus were additionally activated in the dual-task rather than in the single component tasks in healthy controls. These three areas were chosen as the regions of interest (ROIs) for functional connectivity analysis. The ROIs were centered at the voxels showing the maximum magnitude of activation within these regions, with a radius of 5 mm. We only chose the smoothed data at task conditions for connectivity analysis. The images were filtered with a high-pass filter (128 seconds). We measured functional connectivity with partial correlation analysis by a toolkit REST (www.restfmri.net). The white matter and cerebrospinal fluid signals were regressed out. A seed reference time course was obtained within each ROI. Correlation analysis was carried out between the seed reference and the whole brain in a voxel-wise manner in each ROI. The individual results were entered into a random effect one-sample t-test to determine brain regions showing significant connectivity with each ROI within each group in each condition. Then, a two-sample t-test model was used to explore the difference between patients and normal subjects in each condition. Finally, we used one-way ANOVA to examine if there were any brain regions that were additionally connected with the ROIs in the dual-task compared to single tasks in each group. A family-wise error (FWE) corrected threshold of P < 0.05 was used for all brain activity and connectivity analysis. Extent threshold was 10 voxels.

## Additional Information

**How to cite this article**: Gao, L. *et al*. The cerebellum in dual-task performance in Parkinson’s disease. *Sci. Rep.* **7**, 45662; doi: 10.1038/srep45662 (2017).

**Publisher's note:** Springer Nature remains neutral with regard to jurisdictional claims in published maps and institutional affiliations.

## Supplementary Material

Supplementary Tables and Figures

## Figures and Tables

**Table 1:** Brain regions additionally activated in dual-task.

Group	Brain region	Brodmann area	MNI coordinates			t value	Cluster size (mm^3^)
			x	y	z		
Controls	R Precuneus	7	3	−57	51	5.84	594
	L Cerebellum, Anterior Lobe, Culmen, Lobule V		−27	−39	−23	6.17	899
	R Cerebellum, Vermis		3	−51	−15	6.51	972
PD patients	Precuneus	7	0	−51	51	5.72	675

Brain regions additionally activated during performance of dual-task compared with tapping and counting tasks in each group. Abbreviations: L, left; and R, right.

**Table 2:** Brain regions more activated in PD patients compared to controls.

Task	Brain region	Brodmann area	MNI coordinates			t value	Cluster size (mm^3^)
			x	y	z		
Tapping	L M1	4	−30	−33	63	8.01	918
	R PMC	6	48	2	51	7.29	378
	L Superior Parietal Lobule	7	−18	−69	51	7.67	621
	L Inferior Parietal Lobule	40	−27	−54	57	7.88	351
	L Cerebellum, Anterior Lobe, Culmen, lobule V		−26	−45	−27	7.31	486
	R Cerebellum, Anterior Lobe, Vermis		3	−50	−16	8.07	891
	R Cerebellum, Posterior Lobe, Declive, lobule VI		23	−65	−25	7.83	810
	R Cerebellum, Posterior Lobe, Tonsil		28	−44	−42	7.92	513
Dual-task	R Precuneus	5	−6	−45	66	7.89	1215
	R Paracentral Lobule	5	18	−45	57	6.10	432
	R PMC	6	30	−10	54	6.64	1404
	R Prefrontal Cortex	9	33	25	39	6.05	864
	L Postcentral Gyrus	40	−23	−42	50	8.69	1944
	R Cerebellum, Posterior Lobe, Declive, lobule VI		22	−62	−24	8.11	1161
	R Cerebellum, Posterior Lobe, Uvula		32	−76	−32	6.02	594

Abbreviations: L, left; R, right; M1, primary motor cortex; PMC, premotor cortex.

**Table 3:** Demographics and clinical details of the subjects (mean ± SD).

	PD patients	Controls
Age (years)	62.50 ± 6.95 (50–75)	62.28 ± 6.76 (50–74)
Sex	7 female, 11 male	7 female, 11 male
Disease Duration (years)	4.17 ± 1.86	
UPDRS motor score	18.94 ± 5.84	
Hoehn and Yahr staging	1.64 ± 0.59	
Mini-Mental State Examination	29.06 ± 1.11	29.28 ± 0.96
L-dopa dose (mg/day)	347.22 ± 101.06	

The comparisons between the PD patients and controls were analyzed with two-sample t-tests (p > 0.05).
