# Supplementary material for: The cerebellum in dual-task performance in Parkinson’s disease
Source: Sci Rep. 2017 Mar 30;7:45662. doi: 10.1038/srep45662 (PMC5372469; doi:10.1038/srep45662)
Supplement: Supplementary Tables and Figures [file srep45662-s1.pdf]

The cerebellum in dual-task performance in Parkinson's disease

Linlin Gao[1,2], Jiarong Zhang[1,2], Yanan Hou[1,2], Mark Hallett[3], Piu Chan[1,2], Tao Wu[1,2],

[1]Department of Neurobiology, Key Laboratory on Neurodegenerative Disorders of Ministry of Education, Beijing Institute of Geriatrics, Xuanwu Hospital, Capital Medical University, Beijing, China

[2]Beijing Key Laboratory on Parkinson's Disease, Parkinson Disease Center of Beijing Institute for Brain Disorders, Beijing, China

[3]Human Motor Control Section, Medical Neurology Branch, National Institute of Neurological Disorders and Stroke, National Institutes of Health, Bethesda, MD, USA

Correspondence to:

Tao Wu, MD, PhD

Department of Neurobiology, Key Laboratory on Neurodegenerative Disorders of Ministry of Education, Beijing Institute of Geriatrics, Xuanwu Hospital, Capital Medical University, Beijing, 100053, China

Tel: 86-10-8319-8271

Fax: 86-10-8316-1294

E-mail: wutao69@gmail.com

Word count: Abstract: 169; Text: 4221

Tables: 3; Figures: 5

Running Title: Dual-task performance in PD

Keywords: Parkinson's disease; dual-task; cerebellum; brain resources; neural

networks

Financial Disclosure: nothing to disclose

Funding sources for study: This work was supported by grant from the National Science Foundation of China (81571228).

Author Roles:

Linlin Gao: execution of the research, analysis of the data, writing of the manuscript;

Jiarong Zhang, Yanan Hou: execution of the research, analysis of the data;

Mark Hallett, Piu Chan: conception of the research, advice on data analysis;

Tao Wu: conception, organization of the research, and critique of the manuscript.

Supplementary Figure 1. Brain deactivations in PD patients and controls

Brain regions deactivated during performing the dual-task in the healthy control group (A), and in PD patients (B). one sample t-test, $P < 0.05$, FWE corrected. T-value bars are shown on the right.

Supplementary Figure 2. Brain regions connected with the left lobule V of the cerebellum (LCV)

Brain regions functionally connected with the LCV during performing the right hand finger tapping task (A), counting task (B) and dual-task (C) in the healthy control group (left column), and in PD patients (right column). one sample t-test, $P < 0.05$, FWE corrected. T-value bars are shown on the right.

Supplementary Figure 3. Brain regions connected with the precuneus

Brain areas connected with the precuneus in the healthy control group (A) and in PD patients (B), and brain regions more connected with the precuneus in PD patients than in controls (C) during performance of dual-task. T-value bars are shown on the right.

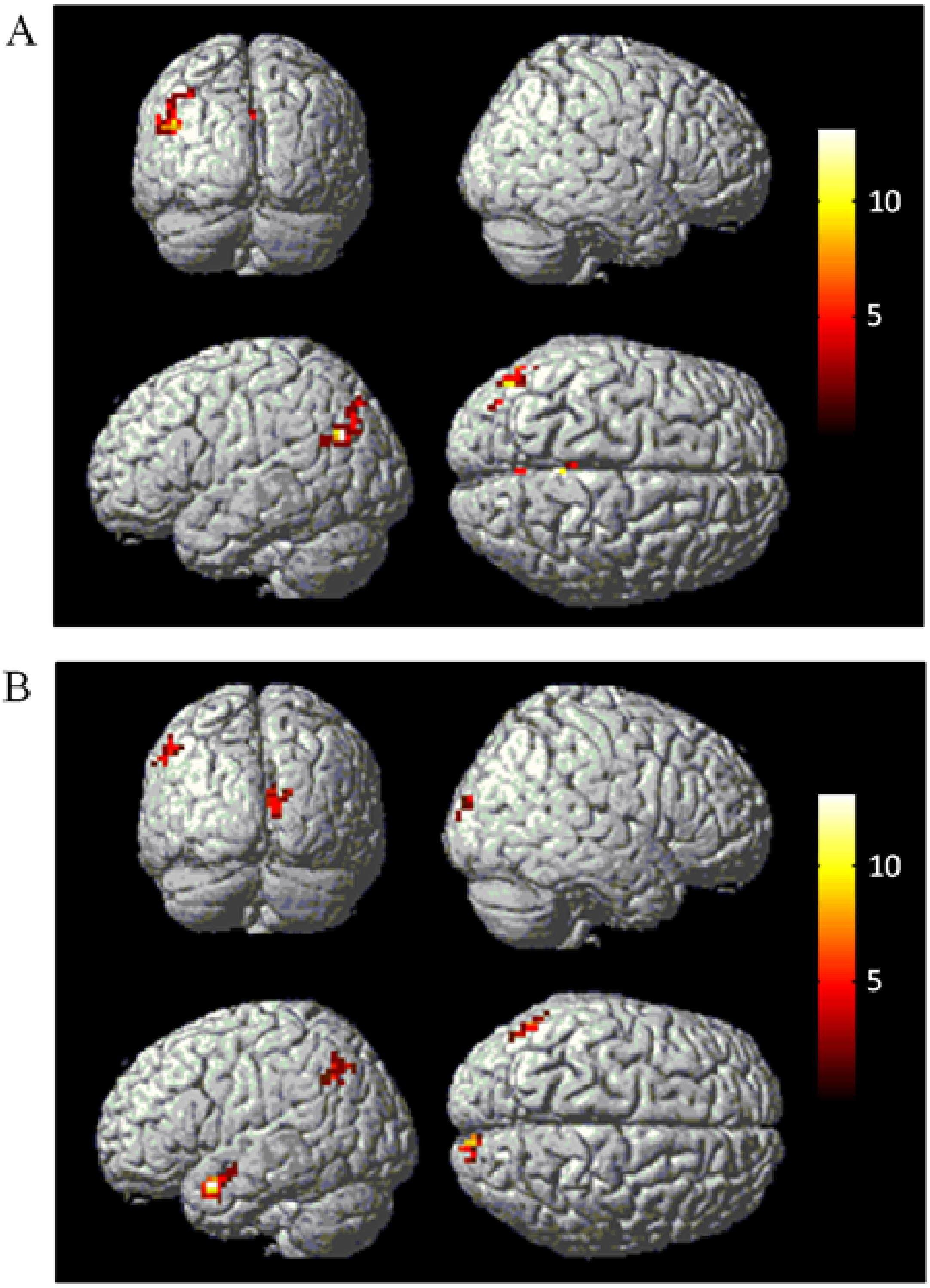

Supplementary Figure 1. Brain deactivations in PD patients and controls

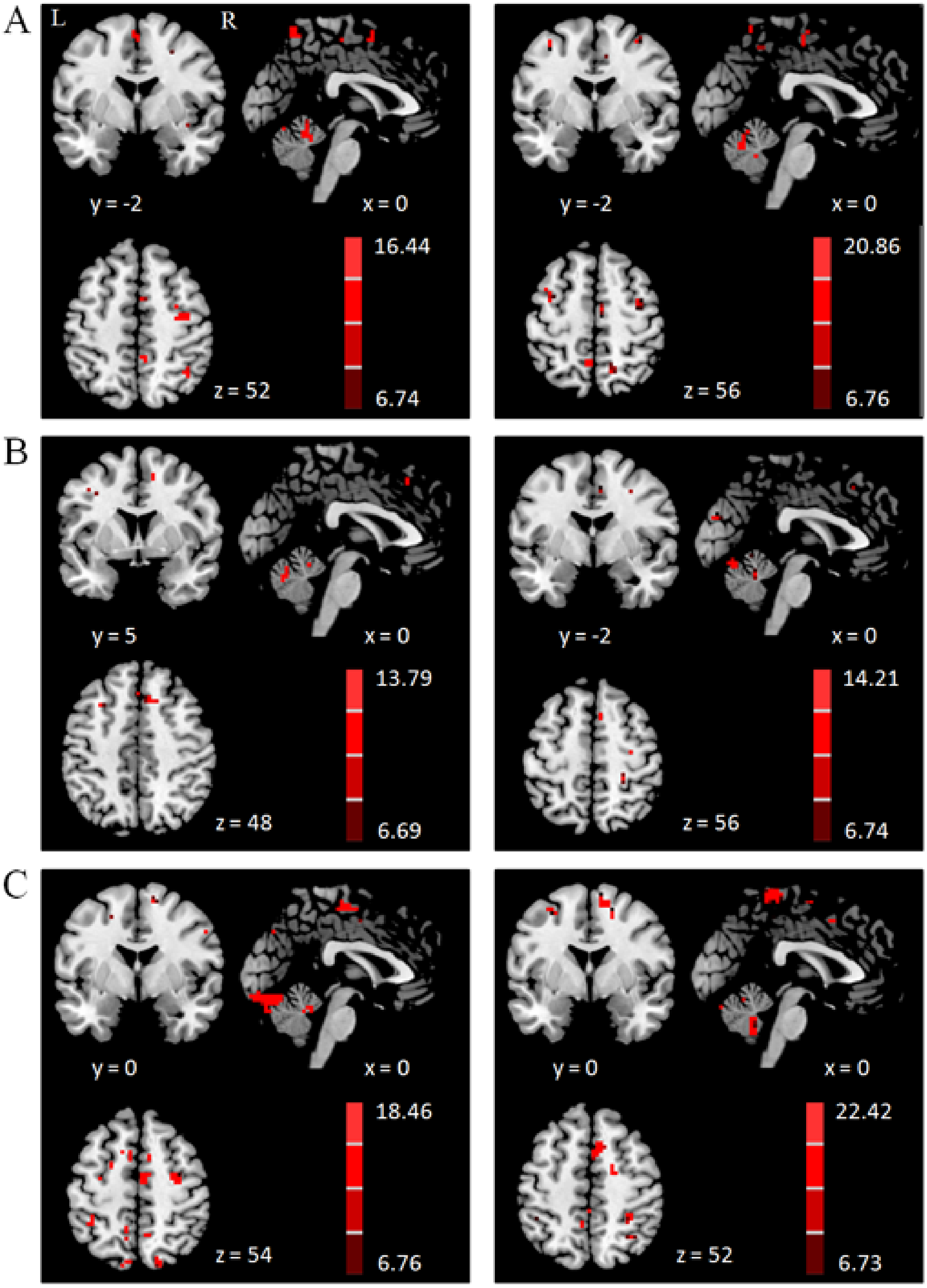

Supplementary Figure 2. Brain regions connected with the left lobule V of the cerebellum (LCV)

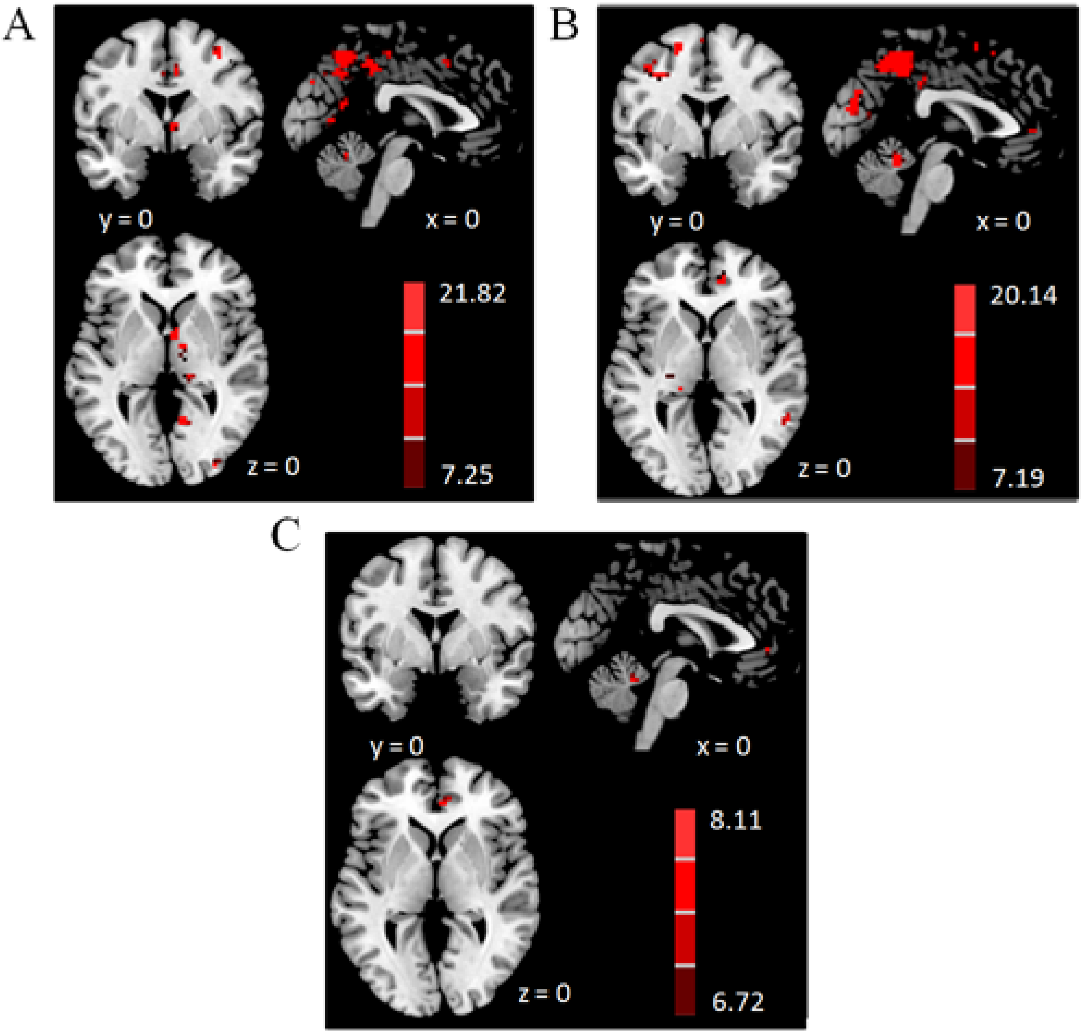

Supplementary Figure 3. Brain regions connected with the precuneus

Supplementary Table 1. Connectivity in the RVM in controls

Task	Brain region	Brodmann area	MNI coordinates x	y	z	t value	Cluster size (mm^3)
Tapping							
	L M1	4	-44	-22	54	8.64	567
	L PMC	6	-28	-9	69	11.36	972
	R SMA-proper	6	3	-6	60	10.90	1809
	L Cerebellum, Anterior Lobe, Culmen		-18	-57	-27	8.93	324
	L Cerebellum, Posterior Lobe, Declive		-24	-75	-24	10.27	729
	R Cerebellum, Anterior Lobe		27	-45	-36	9.65	594
	R Cerebellum, Vermis		4	-61	-16	12.76	2241
Counting							
	L PMC	6	-27	-4	51	8.48	297
	R PMC	6	36	-3	45	8.82	270
	R Pre-SMA	6	9	3	48	8.38	486
	L Cerebellum, Posterior Lobe, Declive		-24	-54	-18	10.94	405
	R Cerebellum, Posterior Lobe, Declive		33	-60	-27	7.92	567
	R Cerebellum, Vermis		3	-60	-15	11.50	2943
Dual-task							
	L Postcentral Gyrus	2	-54	-24	51	10.51	270
	R Postcentral Gyrus	2	48	-30	39	10.95	432
	L M1	4	-36	-23	54	9.26	1377
	L PMC	6	-29	-9	57	15.85	3051
	L Pre-SMA	6	-3	13	51	16.92	5076
	L SMA-proper	6	-7	-11	69	10.39	270
	R PMC	6	38	-6	48	13.89	2403
	L Precuneus	7	-12	-75	45	10.17	351
	R Inferior Parietal Lobule	40	42	-39	48	11.63	864
	L Inferior Parietal Lobule	40	-30	-48	42	9.67	297
	L Cerebellum, Posterior Lobe, Declive		-36	-69	-27	12.95	2619
	R Cerebellum, Posterior Lobe, Declive		36	-72	-24	12.70	1917
	R Cerebellum, Vermis		6	-61	-18	16.00	6588
	R Thalamus		12	-9	9	10.34	324

Abbreviations: L, left; R, right; M1, primary motor cortex; PMC, premotor cortex; pre-SMA, rostral supplementary motor area; SMA-proper, caudal supplementary motor area

Supplementary Table 2. Connectivity in the RVM in PD patients

Task	Brain region	Brodmann area	MNI coordinates x	y	z	t value	Cluster size (mm^3)
Tapping							
	R Postcentral Gyrus	2	51	-21	42	10.24	2430
	L M1	4	-34	-26	56	11.46	5049
	L PMC	6	-36	-3	54	13.26	17010
	L Pre-SMA	6	-6	6	51	11.53	1809
	R PMC	6	33	-4	54	12.21	3213
	SMA-proper	6	-8	-6	53	10.34	4212
	L Precuneus	7	-21	-75	42	14.37	4698
	R Precuneus	7	15	-60	57	10.43	2457
	R Prefrontal Cortex	9	39	36	30	10.53	1134
	R Middle Temporal Gyrus	21	51	-33	-6	9.82	1377
	R Inferior Parietal Lobule	40	42	-48	54	9.07	378
	L Middle Frontal Gyrus	46	-42	39	24	10.74	1161
	L Cerebellum, Posterior Lobe, Declive		-24	-63	-27	14.58	7695
	R Cerebellum, Vermis		-6	-63	-16	16.33	23139
Counting							
	L Pre-SMA	6	-3	9	64	8.22	378
	L PMC	6	-33	-1	48	7.66	324
	R PMC	6	39	0	51	9.07	297
	L Cerebellum, Anterior Lobe, Culmen		-24	-54	-21	11.79	2457
	L Cerebellum, Posterior Lobe, Declive		-12	-81	-21	9.33	270
	R Cerebellum, Vermis		-4	-60	-20	15.38	9153
Dual-task							
	R Postcentral Gyrus	2	66	-24	27	10.86	459
	L M1	4	-30	-29	58	12.05	2754
	R M1	4	34	-24	55	12.43	2970
	L SMA-proper	6	-6	-16	57	11.87	2754
	R PMC	6	41	0	54	10.27	1431
	L Pre-SMA	6	-3	14	57	12.24	1674
	L PMC	6	-26	-3	55	10.34	2943
	R Precuneus	7	6	-72	47	15.03	432
	L Superior Parietal Lobule	7	-19	-59	61	12.20	2484
	L Occipital Lobe	19	-27	-75	30	13.13	1377
	R Inferior Temporal Gyrus	20	63	-30	-21	11.36	1080
	R Middle Temporal Gyrus	21	63	-51	-9	10.77	513
	R Superior Temporal Gyrus	21	66	-42	3	9.66	324
	L Superior Temporal Gyrus	22	-60	-39	12	9.58	324
	L Inferior Parietal Lobule	40	-36	-42	48	12.29	3483

R Inferior Parietal Lobule	40	41	-47	52	9.88	1836
L Postcentral Gyrus	40	-30	-39	56	11.11	3375
L Cerebellum, Posterior Lobe, Declive		-23	-64	-28	12.12	18657
L Thalamus, Ventral Lateral Nucleus		-15	-16	9	11.26	1701
R Cerebellum, Posterior Lobe, Declive		21	-56	-24	15.20	13473
R Cerebellum, Posterior Lobe, Pyramis		12	-78	-36	9.62	459
R Cerebellum, Vermis		6	-62	-18	16.84	14769

Abbreviations: L, left; R, right; M1, primary motor cortex; PMC, premotor cortex; pre-SMA, rostral supplementary motor area; SMA-proper, caudal supplementary motor area

Supplementary Table 3. Connectivity in the LCV in controls

Task	Brain region	Brodmann area	MNI coordinates x	y	z	t value	Cluster size (mm^3)
Tapping							
	R SMA-proper	6	3	-4	60	8.94	324
	R PMC	6	36	-9	51	8.83	783
	L Precuneus	7	-6	-63	63	9.25	2106
	R Insula	13	40	2	-9	8.12	378
	R Inferior Parietal Lobule	40	36	-54	48	9.13	864
	L Cerebellum, Posterior Lobe, Tonsil		-36	-57	-51	8.37	459
	L Cerebellum, Anterior Lobe, Culmen		-28	-42	-19	12.44	6156
	R Cerebellum, Anterior Lobe, Culmen		19	-40	-21	10.68	5238
Counting							
	R Pre-SMA	6	9	17	48	8.84	351
	L PMC	6	-36	-9	48	8.30	405
	L Cerebellum, Anterior Lobe, Culmen		-25	-43	-22	12.79	4509
	R Cerebellum, Posterior Lobe, Declive		33	-66	-21	10.06	3537
Dual-task							
	R Pre-SMA	6	9	18	45	8.92	297
	SMA-proper	6	0	-13	60	9.87	1485
	L PMC	6	-21	-9	60	8.14	297
	R PMC	6	30	-6	54	8.61	621
	L Precuneus	7	-8	-66	49	8.18	864
	R Precuneus	7	11	-61	44	8.10	378
	L Middle Temporal Gyrus	37	-54	-54	-3	8.91	891
	L Inferior Parietal Lobule	40	-35	-45	54	9.06	594
	L Cerebellum, Anterior Lobe, Culmen		-33	-57	-33	11.73	3294
	L Cerebellum, Posterior Lobe, Declive		-28	-65	-24	10.46	1998
	R Cerebellum, Anterior Lobe, Culmen		22	-41	-26	10.66	4617
	R Cerebellum, Posterior Lobe, Declive		37	-64	-24	12.11	5805

Abbreviations: L, left; R, right; PMC, premotor cortex; pre-SMA, rostral supplementary motor area; SMA-proper, caudal supplementary motor area

Supplementary Table 4. Connectivity in the LCV in PD patients

Task	Brain region	Brodmann area	MNI coordinates x	y	z	t value	Cluster size (mm^3)
Tapping							
	R Cerebellum, Anterior Lobe, Culmen		15	-42	-18	10.20	6534
	R Cerebellum, Posterior Lobe, Tonsil		39	-60	-39	8.39	432
	L Cerebellum, Anterior Lobe, Culmen		-24	-44	-24	12.86	6453
	L Cerebellum, Posterior Lobe, Declive		-36	-62	-27	10.64	3051
	R SMA-proper	6	6	-14	49	8.79	945
	L PMC	6	-39	2	57	9.25	351
	R PMC	6	36	-6	60	8.12	270
	R Precuneus	7	9	-45	69	8.54	756
Counting							
	R Pre-SMA	6	6	12	57	8.90	810
	R PMC	6	36	-6	58	8.12	270
	R Postcentral Gyrus	3	26	-33	57	8.52	486
	L Precuneus	31	-8	-77	29	9.04	756
	L Cerebellum, Anterior Lobe, Culmen		-30	-45	-22	13.21	3618
	R Cerebellum, Posterior Lobe, Declive		9	-78	-27	9.97	648
	R Cerebellum, Anterior Lobe, Culmen		18	-45	-15	12.46	2997
Dual-task							
	L SMA-proper	6	-6	-13	67	8.17	297
	L PMC	6	-32	-5	60	8.22	405
	R pre-SMA	6	6	18	48	9.41	1053
	R Paracentral Lobule	5	3	-39	66	10.44	1728
	R Precuneus	7	6	-57	66	10.27	729
	R PMC	6	26	-4	57	9.04	432
	R Inferior Parietal Lobule	40	33	-45	48	8.57	378
	L Inferior Parietal Lobule	40	-42	-44	47	8.36	270
	R Superior Parietal Lobule	7	32	-56	56	9.11	324
	L Cerebellum, Anterior Lobe, Culmen		-25	-41	-22	12.68	9396
	L Cerebellum, Posterior Lobe, Declive						3699
	R Cerebellum, Anterior Lobe, Culmen						1917
